# Comparison of clinical and radiological outcomes in intertrochanteric fractures treated with InterTAN nail against conventional cephalomedullary nails: a systematic review

**DOI:** 10.2144/fsoa-2020-0182

**Published:** 2020-12-07

**Authors:** Akshay Date, Mrinalini Panthula, Anita Bolina

**Affiliations:** 1 Basildon and Thurrock University Hospital, Nethermayne, Basildon, Essex, SS16 5NL, UK; 2 Imperial College London, Exhibition Road, South Kensington, London SW7 2BU, UK

## Abstract

Intertrochanteric fractures, accountable for 50% of hip fractures, can be fixed with cephalomedullary devices such as Proximal Femoral Nail Antirotation (PFNA™), Gamma3 nailing system and TRIGEN™ InterTAN™ nail (IT). IT uniquely uses two cephalocervical screws that allow for linear compression and provide additional resistance to femoral head rotation. A literature review assessing clinical outcomes of these devices was conducted, with 14 studies enrolling 3104 patients meeting the inclusion criteria. PFNA and Gamma3 had better intraoperative outcomes compared with IT; however, IT had superior implant-related outcomes of cut-out and screw migration. No difference was found between IT and PFNA or Gamma3 in Harris Hip Scores, time to union, malunion and nonunion. Further long-term studies are needed to evaluate clinical outcomes and cost–effectiveness of cephalomedullary devices.

## Lay abstract

Hip fractures remain a significant cause of morbidity and mortality within healthcare systems, with an estimated global incidence of 1.6 million fractures annually. Certain intertrochanteric fractures – a specific subtype of hip fractures – are treated surgically using intramedullary nailing devices such as Proximal Femoral Nail Antirotation (PFNA™), Gamma3 nailing system and InterTAN™ nail (IT). IT is unique, in that it uses two proximal screws. This study assessed the clinical and radiological outcomes of using IT over the traditional one-screw devices PFNA and Gamma3.

Hip fractures are common orthopedic injuries, with an estimated annual incidence of over 1.6 million fractures globally [1,2]. In the UK, the healthcare and social costs of hip fractures total over GBP£2 billion annually [2]. Hip fractures are associated with significant morbidity and carry a high 30-day postoperative mortality rate of 6.1% [3]. It is therefore important to consider operative and postoperative implications of surgical techniques used for fracture fixation.

Intertrochanteric fractures account for up to half of hip fractures and are usually seen in the elderly demographic, resulting from low-energy injuries [4]. Surgical procedures in intertrochanteric fractures are primarily influenced by fracture pattern. Guidelines recommend the use of sliding hip screws to treat stable fractures, whereas subtrochanteric or unstable fractures are managed with an intramedullary device [4,5]. Intramedullary devices include the Proximal Femoral Nail Antirotation (PFNA™ , IN, USA) nail, Gamma3 (MI, USA) nailing system and TRIGEN™ InterTAN™ (London, UK) nail (IT).

IT differs from traditional intramedullary devices, in that it uniquely uses two cephalocervical screws that allow for linear compression and provide additional resistance to femoral head rotation. Biomechanical studies in cadaveric models have found IT to provide greater biomechanical stability compared with single-screw devices [6–8]. IT has also been shown in cadavers to have significantly greater biomechanical strength, with reduced varus collapse and femoral head rotation, compared with the PFNA and Gamma3 nails [7,8]. Recent literature with regard to clinical studies also suggests the newer generation IT may offer additional rotational stability while reducing postoperative pain, implant failure and nonunion [9]. This systematic review aims to evaluate whether InterTAN offers better clinical or radiological outcomes compared with conventional PFNA and Gamma3 nails for the surgical treatment of intertrochanteric fractures.

## Methods

### Literature search

A systematic review of the literature was performed using the following databases: PubMed, Medline (1946–present) and Embase (1947–31 July 2020). The search aimed to identify original articles comparing IT with either Gamma3 nail or PFNA in patients with intertrochanteric fractures, with a specific focus on intraoperative and postoperative outcomes. Three strings were applied using the following search terms: intertrochanteric, gamma3, PFNA and PFNA II. The Boolean operators ‘AND’ and ‘OR’ were used, and all variations in spelling were included. Reference lists of selected articles were also searched to identify any other relevant studies.

### Eligibility criteria

All articles published up to and including July 2020 were considered. Two independent reviewers (AD, MP) screened the abstracts and titles of articles identified through primary electronic search. Abstracts of eligible articles were obtained for assessment of inclusion criteria. Inclusion criteria were studies in the English language, publications from any country, human studies, studies on intertrochanteric fractures, intraoperative or postoperative outcomes assessed and IT compared with either Gamma3 or PFNA. All randomized controlled trials and both prospective and retrospective observational studies were included. Exclusion criteria were case reports, systematic reviews and meta-analyses, non-human studies, studies in non-English languages, studies not assessing outcomes and studies evaluating only sliding hip screw devices.

### Assessment of quality of studies

Full text articles were obtained of all included studies and were screened by two independent reviewers (AD, MP) for quality. All studies were evaluated using the quality assessment of case–control series screening tool from the National Heart, Lung and Blood Institute [10]. All studies were assessed using this tool's nine-point criteria, and a study's quality rating was graded as good, fair or poor based on total score.

### Outcome measures

Data on both intraoperative and postoperative outcomes were extracted from the included articles. Data included study characteristics (year of publication, sample size, length of follow-up), patient characteristics, intraoperative data (fluoroscopy time, operation time, blood loss), Harris Hip Score (HHS), time to union, periprosthetic fractures, implant failures, delayed union or nonunion and postoperative outcomes (pain, length of stay in hospital and complications). HHS is a clinical score that evaluates different methods of treatment by looking at pain, function, absence of deformity and range of motion, with the results being interpreted as follows: <70 = poor, 70–80 = fair, 80–90 = good and 90–100 = excellent [11].

## Results

### Study characteristics

**Figure 1.**
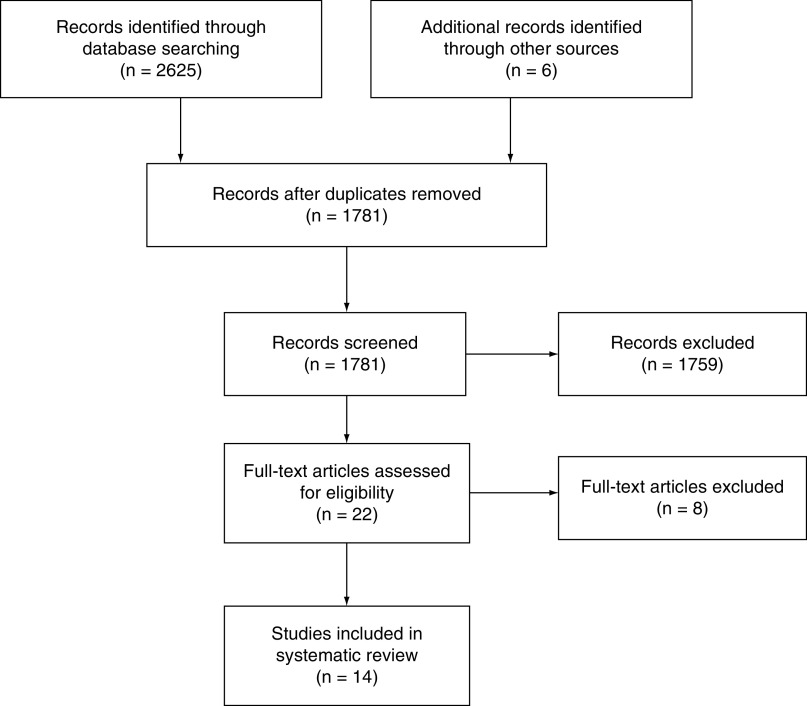
PRISMA chart.

Initial electronic searches identified 2625 articles, of which 14 were eligible for inclusion in this study (Figure 1). All the studies scored ‘good’ on the National Heart, Lung and Blood Institute criteria and were thus included for the scope of this review. Together, the included papers had data on a total of 3104 patients undergoing surgery for intertrochanteric fractures, of which 2683 were available for follow-up and assessment of postoperative outcomes. Ten studies were retrospective case series, two studies were prospective case series and two were randomized controlled trials (Table 1). The mean age of patients in the included studies was 73.3 years, and the average follow-up time was 25.0 months.

**Table 1.** Study characteristics of included studies.

Study (year)	Study design	Devices compared	Patients (n)	Followed up (n)	Mean follow-up time (months)	Mean age (years)	Male patients (%)	Ref.
Wu *et al.* (2014)	Retrospective	IT and Gamma3	261	261	12.0	72.0	23.9	[16]
Yu *et al.* (2016)	Retrospective	IT and PFNA	176	147	20.0	74.5	45.5	[13]
Serrano *et al.* (2017)	Retrospective	IT and Gamma3	463	463	12.0	76.0	33.0	[25]
Makki *et al.* (2015)	Retrospective	IT and PFNA	58	58	NA^†^	79.0	30.0	[22]
Hopp *et al.* (2016)	Prospective	IT and Gamma3	78	60	5.9	81.7	33.3	[17]
Duramaz *et al.* (2019)	Retrospective	IT and PFNA	303	303	12	61.3	43.6	[14]
Zehir *et al.* (2015)	Retrospective	IT and PFNA	276	265	16.0	77.0	38.4	[12]
Imerci *et al.* (2018)	Retrospective	IT and PFNA	NA	69	12.0	56.3	60.9	[23]
Seyhan *et al.* (2015)	Prospective	IT and PFNA	88	75	12.0	75.7	25.3	[15]
Hui Zhang *et al.* (2017)	Retrospective	IT and PFNA	283	239	38.8	76.1	58.9	[19]
Hui Zhang *et al.* (2017) (2)	Retrospective	IT and PFNA	243	174	40	73.0	36.7	[21]
Su *et al.* (2016)	Randomized controlled trial	IT and Gamma3	100	86	12	70.7	40.0	[18]
Berger-Groch *et al.* (2016)	Randomized controlled trial	IT and Gamma3	104	33	60	81.2	23.1	[24]
Chi Zhang *et al.* (2018)	Retrospective	IT and PFNA	417	326	43.3	72.3	46.0	[20]

† Variable: patients followed up until satisfactory union.

IT: InterTAN; NA: Not applicable; PFNA: Proximal femoral nail antirotation.

### Intraoperative outcomes

Intraoperative outcomes reported in the studies included fluoroscopy time (n = 1104), operation time (n = 2086) and blood loss (n = 1633) (Table 2). Five studies reported fluoroscopy time, out of which three found that IT took statistically significantly longer than PFNA (p < 0.001; p < 0.05; p < 0.001) [12–14]. Seyhan *et al.* did not find significance (p = 0.749) between IT and PFNA fluoroscopy times [15]. Wu *et al.* found that IT also had greater fluoroscopy time than Gamma3 (p = 0.012) [16].

**Table 2.** Impact of InterTAN and PFNA or Gamma3 on intraoperative outcomes of fluoroscopy time (n = 1104), operation time (n = 2086) and blood loss (n = 1633).

Study (year)	Patients (n)	Mean age (years)	InterTAN	PFNA	p-value	Ref.
**Fluoroscopy time**			**Fluoroscopy time (mins)**	**Fluoroscopy time (mins)**		
Zehir *et al*. (2015)	276	77.0	2.0	1.5	**< 0.001**^‡^	[12]
Yu *et al.* (2016)	176	74.5	5.0	2.8	**< 0.05**	[13]
Seyhan *et al.* (2015)	88	75.7	1.0	1.1	0.749	[15]
Wu *et al.* (2014)	261	72.0	2.9	2.6^†^	**0.012**	[16]
Duramaz *et al*. (2019)	303	61.3	34.6	29.9	**< 0.001**^§^	[14]
**Operation time**			**Operation time (mins)**	**Operation time (mins)**		
Zehir *et al*. (2015)	276	77.0	55.4	44.4	**< 0.001**^‡^	[12]
Yu *et al.* (2016)	176	74.5	71.9	52.3	**< 0.05**	[13]
Seyhan *et al*. (2015)	88	75.7	73.9	73.0	0.721	[15]
Zhang *et al*. (2017)	283	76.1	67.2	68.9	0.848	[19]
Zhang *et al*. (2018)	417	72.3	65.8	66.2	0.216	[20]
Berger-Groch *et al*. (2016)	104	81.2	48.0	51.0^†^	0.52	[24]
Hopp *et al.* (2016)	78	81.7	78.0	64.6^†^	**0.044**	[17]
Su *et al*. (2016)	100	70.1	52.3	66.7^†^	**0.034**	[18]
Wu *et al.* (2014)	261	72.0	63.7	59.9^†^	**0.011**	[16]
Duramaz *et al.* (2019)	303	61.3	61.6	52.5	**< 0.001**^§^	[14]
**Blood loss**			**Blood loss (ml)**	**Blood loss (ml)**		
Zehir *et al.* (2015)	276	77.0	211.4	139.7	**< 0.001**^‡^	[12]
Yu *et al.* (2016)	176	74.5	190.6	180.9	**< 0.05**	[13]
Zhang *et al*. (2017)	283	76.1	180.7	185.1	0.078	[19]
Zhang *et al*. (2018)	417	72.3	198.9	199.4	0.092	[20]
Hopp *et al.* (2016)	78	81.7	168.1	175.7^†^	0.915	[17]
Su *et al*. (2016)	100	70.1	80.3	130.6^†^	**0.025**	[18]
Duramaz *et al*. (2019)	303	61.3	204.2	196.9	**0.012**	[14]
Wu *et al.* (2014)	261	72.0	87	86^†^	0.181	[16]

Bold font indicates p-values with statistical significance.

† Gamma3 nail.

‡ Includes comparison of InterTAN with both PFNA and Talon™ lag screw.

§ Includes comparison of InterTAN with both PFNA and Profin^®^.

PFNA: Proximal femoral nail antirotation.

Six studies compared differences in operating time between IT and PFNA, and four compared IT with Gamma3. Three out of the six studies reported that the IT cohort had a significantly longer operation time compared with PFNA (p < 0.001; p < 0.001; p < 0.05) [12–14]. Of studies looking at Gamma3, two found that IT had a significantly longer operation time compared with Gamma3 (p = 0.044; p = 0.011) [16,17]. By contrast, one study found Gamma3 had a significantly longer operation time (p = 0.034) [18].

Five studies [12–14,19,20] reported the impact of IT compared with PFNA on blood loss, and three [16–18] compared IT with Gamma3. Three studies reported that patients with IT had significantly increased blood loss compared with those with PFNA (p < 0.001; p < 0.05; p = 0.012) [12–14]. Two studies found that IT had less blood loss than Gamma3, although only one found this significant (p = 0.025) [17,18].

### Postoperative outcomes

#### Harris Hip Score

Twelve studies reported HHS (n = 2038) in patients, of which eight compared IT with PFNA and one compared IT with Gamma3 (Table 3). Two studies found that IT had a significantly higher HHS compared with PFNA (p < 0.001; p = 0.038) [14,21]. There was no significant difference in HHS reported between patients treated with IT compared with PFNA or Gamma3 in the remaining studies.

**Table 3.** Impact of InterTAN and PFNA or Gamma3 on HHS (n = 2038).

Study (year)	Total patients (n)	Patients followed up (n)	Mean age (years)	Mean follow-up time (months)	HHS, InterTAN	HHS, PFNA	p-value	Ref.
Zehir *et al*. (2015)	276	265	77.0	16.0	71.3	75.9	0.32^‡^	[12]
Seyhan *et al*. (2015)	88	75	74.5	12.0	82.4	80.9	0.11	[15]
Imerci *et al*. (2018)	NA	69	56.3	12.0	82.6	79.8	0.294	[23]
Yu *et al.* (2016)	176	147	74.5	20.0	82.6	83.8	0.302	[13]
Hui Zhang *et al*. (2017)	283	239	76.1	38.8	72.2	72.4	NA	[19]
Hui Zhang *et al*. (2017) (2)	243	174	73	40	75.1	71.0	**< 0.001**	[21]
Chi Zhang *et al*. (2018)	417	326	72.3	43.3	80.2	79.7	0.187	[20]
Berger-Groch *et al*. (2016)	104	33	81.2	60	69.6	70.4^†^	0.84	[24]
Hopp *et al*. (2016)	78	60	81.7	5.9	42.8	35.3^†^	0.298	[17]
Su *et al*. (2016)	100	86	70.7	12	63.3	64.7^†^	0.136	[18]
Duramaz *et al*. (2019)	303	303	61.3	12	75.2	76.8	**0.038**^§^	[14]
Wu *et al.* (2014)	261	261	72.0	12	88.2	85.6^†^	0.076	[16]

Bold font indicates p-values with statistical significance.

† Gamma3 nail.

‡ Includes comparison of InterTAN with both PFNA and Talon™ lag screw.

§ Includes comparison of InterTAN with both PFNA and Profin^®^.

HHS: Harris Hip Score; NA: Not applicable; PFNA: Proximal femoral nail antirotation.

#### Time to union

With regard to time to union, three studies compared IT with PFNA, and one study compared IT with Gamma3 (n = 556) (Table 4). There was no statistically significant difference reported in the time to union between IT and PFNA or Gamma3.

**Table 4.** Impact of InterTAN compared with PFNA on time to union (n = 556).

Study (year)	Total patients (n)	Patients followed up (n)	Mean age (years)	Mean follow-up time (months)	InterTAN, n (weeks)	PFNA (weeks)	p-value	Ref.
Seyhan *et al*. (2015)	88	75	75.7	12.0	9.9	9.79	0.871	[15]
Imerci *et al*. (2018)	NA	69	56.3	12.0	17.1	17.8	0.573	[23]
Chi Zhang *et al*. (2018)	417	326	72.3	43.3	14.8	15.2	NA	[20]
Su *et al*. (2016)	100	86	70.7	12	15.2	14.9^†^	0.286	[18]

Bold font indicates p-values with statistical significance.

† Gamma3 nail.

NA: Not applicable; PFNA: Proximal femoral nail antirotation.

#### Delayed union, malunion and nonunion

Six studies compared the number of cases of delayed union, malunion or nonunion between IT and PFNA (n = 852) (Table 5). Zhang *et al.* reported the number of cases of delayed union, malunion and nonunion and found that there was no statistically significant difference between IT and PFNA across all three groups [21]. Similarly, three studies looking at nonunion rates found no significant difference between IT and PFNA groups [15,22,23]. Two studies looked at IT compared with Gamma3, with no significant difference found in rates of nonunion [16,17].

**Table 5.** Impact of InterTAN and PFNA on number of cases of delayed union, malunion and nonunion (n = 852).

Study (year)	Total patients (n)	Patients followed up (n)	Mean age (years)	Mean follow-up time (months)	Complication	InterTAN, n (cases)	PFNA, n (cases)	p-value	Ref.
Hui Zhang *et al*. (2017)	283	329	76.1	38.8	Delayed union	4	5	0.709	[19]
					Malunion	2	0	0.498	
					Nonunion	2	0	0.498	
Makki *et al*. (2015)	58	58	79	NA^†^	Nonunion	2	11	0.1	[22]
Seyhan *et al.* (2015)	88	75	74.5	12.0	Nonunion	0	0	NA	[15]
Hopp *et al*. (2016)	78	60	81.7	5.9	Nonunion	3	3^‡^	0.473	[17]
Imerci *et al*. (2018)	69	69	56.25	12	Nonunion	1	1	NA	[23]
Wu *et al*. (2014)	261	261	72.0	12	Nonunion	1	5^‡^	0.381	[16]

Bold font indicates p-values with statistical significance.

† Variable: patients were followed up until satisfactory union.

‡ Gamma3 nail.

NA: Not applicable; PFNA: Proximal femoral nail antirotation.

#### Pain

Seven studies looked at hip and thigh pain experienced postoperatively by patients (n = 1469), four comparing IT and PFNA and three comparing IT and Gamma3 (Table 6). Two studies reported that patients receiving IT had significantly lower postoperative hip and thigh pain compared with the PFNA group (p = 0.019; p = 0.043) [13,21]. Duramaz and İlter found that a greater percentage of patients with IT experienced pain compared with those with PFNA (29.1 vs 20.0%), although statistical significance was not assessed [14].

**Table 6.** Impact of InterTAN compared with PFNA on postoperative pain (n = 1469).

Study (year)	Patients (n)	Mean age (years)	InterTAN patients reporting postoperative pain (%)	PFNA patients reporting postoperative pain (%)	p-value	Ref.
Zehir *et al*. (2015)^†^	276	77.0	4.9	10.4	0.57^‡^	[12]
Yu *et al*. (2016)^†^	176	74.5	4.8	11.6	**0.019**	[13]
Hui Zhang *et al*. (2017)^†^	283	75.7	3.5	9.4	**0.043**	[19]
Su *et al*. (2016)^†^	92	70.7	6.4	8.9^§^	0.650	[18]
Wu *et al.* (2014)^†^	261	72.0	4.6	4.0^§^	NA	[16]
Duramaz *et al.* (2019)^†^	303	61.3	29.1	20.0	NA	[14]
Hopp *et al*. (2016)^¶^	78	81.7	4.07	4.88^§^	0.169	[17]

Bold font indicates p-values with statistical significance.

† Pain in hip or thigh region reported postoperatively; scoring system not mentioned.

‡ Includes comparison of InterTAN with both PFNA and Talon™ lag screw.

§ Gamma3 nail.

¶ Initial postoperative pain reported using VAS.

NA: Not applicable; PFNA: Proximal femoral nail antirotation; VAS: Visual analog scale.

Two of the studies comparing the percentage of patients experiencing postoperative pain in IT and Gamma3 groups found no statistically significant difference [16,18]. Hopp *et al.* reported postoperative pain using a visual analog scale and found that there was no significant difference between the IT and Gamma3 groups (p = 0.169) [17].

#### Length of hospital stay

Six studies reported the length of time the patient spent in the hospital (n = 1170); three studies compared IT with PFNA, and three studies compared IT with Gamma3 (Table 7). Yu *et al.* found that patients with PFNA had a significantly shorter length of time in the hospital compared with the IT group (p < 0.05) [13], whereas the other two studies reported no significant difference between these groups [12,19]. Berger-Groch *et al.* found a statistically significant shorter length of hospital stay for Gamma3 patients compared with IT patients (p = 0.03) [24], although there was no significant difference between these groups in the other two studies [16,17].

**Table 7.** Impact of InterTAN and PFNA or Gamma3 on length of hospital stay (n = 1170).

Study (year)	Patients (n)	Mean age (years)	InterTAN hospital stay (days)	PFNA hospital stay (days)	p-value	Ref.
Hui Zhang *et al*. (2017)	283	76.1	11.9	11.67	0.21	[19]
Zehir *et al*. (2015)	276	77	7.45	7.14	0.28^‡^	[12]
Yu *et al.* (2016)	168	74.5	9.65	8.58	**< 0.05**	[13]
Berger-Groch *et al*. (2016)	104	81.2	11.6	10.3^†^	**0.03**	[24]
Hopp *et al*. (2016)	78	81.7	16.5	17.8^†^	0.64	[17]
Wu *et al.* (2014)	261	72.0	10.8	11.1^†^	0.081	[16]

Bold font indicates p-values with statistical significance.

† Gamma3 nail.

‡ Includes comparison of IT with both PFNA and Talon™ lag screw.

PFNA: Proximal femoral nail antirotation.

#### Periprosthetic fracture

Five studies looked at periprosthetic fractures with IT and PFNA, and one compared IT and Gamma3 (n = 1312) (Table 8). Wu *et al.* found a significantly greater number of femoral shaft fractures in the Gamma3 group than the IT group (p = 0.044) [16]. Yu *et al.* found a significantly higher number of lateral cortex fractures in patients treated with IT compared with PFNA (p = 0.045) [13]. However, of the remaining four studies comparing IT and PFNA, three found a significantly greater periprosthetic fracture incidence with PFNA than IT [15,19–21]. Zhang *et al.* did not specify fracture type; however, they did report a greater periprosthetic fracture rate in the PFNA group (p = 0.004) [20].

**Table 8.** Impact of InterTAN and PFNA on number of reported cases of secondary periprosthetic fracture (n = 1312).

Study (year)	Total patients (n)	Patients followed up (n)	Mean age (years)	Mean follow-up time (months)	Fracture description	InterTAN	PFNA	p-value	Ref.
Yu *et al.* (2016)	168	147	74.5	20.0	Lateral cortex	8	1	**0.045**	[13]
Seyhan *et al*. (2015)	88	75	75.7	12.0	Nondisplaced	0	1	NA	[15]
					Lateral cortex	0	1	NA	
Chi Zhang *et al.* (2018)	417	326	72.3	43.3	NA	7	22	**0.004**	[20]
Hui Zhang *et al*. (2017)	283	329	76.1	38.8	Femoral shaft	3	12	**0.014**	[19]
Wu *et al.* (2014)	261	261	72.0	12	Femoral shaft	1	10^†^	**0.044**	[16]
Hui Zhang *et al*. (2017) (2)	243	174	73	40	Periprosthetic	0	1	1.000	[21]
					Lateral cortex	0	7	**0.022**	
					Femoral shaft	1	8	**0.044**	

Bold font indicates p-values with statistical significance.

† Gamma3 nail.

NA: Not applicable; PFNA: Proximal femoral nail antirotation.

#### Implant failures

There were seven studies that looked at implant failures in IT and PFNA groups and four studies comparing IT with Gamma3 (n = 2081) (Table 9). Some studies categorized type of failure by group, such as cut-out or lateral screw migration, whereas others did not specify implant failure type. Six studies [12,13,19,21–23] assessed cut-out between IT and PFNA, and four found that PFNA had a significantly higher number of cases compared with IT (p = 0.024; p = 0.033; p = 0.01; p < 0.001) [12,13,19,22]. Three studies [13,21,23] reported screw migration; two studies [13,21] found no statistically significant difference, whereas the remaining study found a significantly higher number of cases in the PFNA group compared with IT (p = 0.008) [12]. Furthermore, Imerci *et al.* found that PFNA patients had a significantly greater reported incidence of shaft fractures, cut-out and lateral migration screw (p = 0.0001) [23].

**Table 9.** Impact of InterTAN and PFNA on number of reported cases of implant failure (n = 2081).

Study (year)	Total patients (n)	Patients followed up (n)	Mean age (years)	Mean follow-up time (months)	Failure type	InterTAN cases (n)	PFNA cases (n)	p-value	Ref.
Hui Zhang *et al*. (2017)	283	239	76.1	38.8	Cut-out	3	11	**0.024**	[19]
Hui Zhang *et al.* (2017) (2)	243	174	73	40	Cut-out	1	2	1.000	[21]
					Generic^‡^	0	6	0.040	
					Screw migration	0	1	1.000	
Yu *et al.* (2016)	168	147	74.5	20	Generic^‡^	3	1	0.641	[13]
					Cut-out	0	6	**0.033**	
					Lateral screw migration	1	1	1.000	
Makki *et al*. (2015)	NA	58	79	NA	Cut-out	0	8	**0.01**	[22]
Imerci *et al*. (2018)	NA	69	56.25	12	Shaft fracture, cut through, lateral screw migration	1	9	0.0001	[23]
Serrano *et al.* (2017)	413	413	76	12	Cut-out and generic^‡^	1	9^†^	**0.007**	[25]
Zehir *et al*. (2015)	276	265	77	16.0	Cut-out	0	8	**< 0.001**^§^	[12]
					Screw migration	0	5	0.008^§^	
Hopp *et al*. (2016)	78	60	81.7	5.9	Secondary dislocation with cut-out	2	1^†^	0.466	[17]
					Secondary varus collapse (no cut-out)	2	3^†^		
Su *et al*. (2016)	100	92	70.7	12	Cut-out	1	5^†^	0.081	[18]
Wu *et al.* (2014)	261	261	72.0	12	Cut-out	1	14^†^	**0.024**	[16]
Duramaz *et al*. (2019)	303	303	61.3	12	Cut-out	4	3	NA	[14]

Bold font indicates p-values with statistical significance.

† Gamma3 nail.

‡ Generic: type of implant failure not specified.

§ Includes comparison of InterTAN with both PFNA and Talon™ lag screw.

NA: Not applicable; PFNA: Proximal femoral nail antirotation.

Two studies compared cut-out on its own between IT and Gamma3, with one study finding significantly more cases in the Gamma3 cohort (p = 0.024) [16]. Serrano *et al.* reported that there was a significantly higher number of cut-outs and ‘catastrophic failures’ in the Gamma3 group compared with the IT group (p = 0.007) [25]. Hopp *et al.* reported no significant difference between the IT and Gamma3 groups with regard to secondary dislocation with cut-out and secondary varus collapse without cut-out (p = 0.466) [17].

#### Other complications

A variety of generic complications were reported by studies and are described here in brief. No studies comparing IT and PFNA found statistical significance in the incidence of postoperative complications [12,15,22]. Zehir *et al.* found no significant differences in pneumonia, pressure ulcers, urinary tract infections, decompensated heart failure, pulmonary embolism, deep vein thrombosis, hematoma or deep and superficial wound infections [12]. Seyhan *et al.* found no significant difference in the number of cases of hematomas between IT and PFNA [15]. Makki *et al.* reported no significant difference in the number of cases of wound infections between IT and PFNA [22].

Of studies looking at Gamma3 and IT, only the investigation by Berger-Groch *et al.* found a significantly higher number of complications in Gamma3 patients compared with IT patients (p = 0.04); these included pneumonia, urinary tract infections, acute kidney failure, hyperglycemic crisis, wound hematoma, wound infection and positional damage [24]. Hopp *et al.* found no statistically significant difference between complication rate in IT patients compared with Gamma3 patients with regard to pulmonary and urinary tract infections (p = 0.797) or hematomas (p = 0.423); however, local surgical debridement was needed in one case in the Gamma3 group [17]. Su *et al.* found no significant difference in pulmonary embolism (p = 0.558) or deep vein thrombosis (p = 0.461) between IT and Gamma3 patients [18]. Wu *et al.* found no significant difference in deep vein thrombosis (p = 0.695), wound hematoma (p = 0.157) or urinary tract infection (p = 0.828) [16].

## Discussion

This systematic review aimed to compare the clinical and radiological outcomes of IT compared with two popular single nail systems, PFNA and Gamma3, in the treatment of intertrochanteric fractures. The authors acknowledge that there are other devices for treating these fractures, including Aesculap^®^ (PA, USA) Targon^®^ (Braun, Germany) PFT and Orthofix Veronail^®^ (TX, USA) which were outside the scope of this review. The authors found that IT was superior to PFNA and Gamma3, with lower implant-related complications of cut-out and screw migration, lower incidence of secondary periprosthetic fractures and less postoperative pain. No differences were found in HHS, time to union or cases of nonunion or malunion. Intraoperatively, both single-screw devices required less fluoroscopy time than IT. Although PFNA had more favorable outcomes in operative time and blood loss than IT, a clear advantage was not seen with Gamma3. PFNA and Gamma3 patients were also shown in some studies to have a lower length of postoperative hospital stay compared with IT.

Intraoperative parameters demonstrated shorter fluoroscopy time using single-screw devices and lower operating times and blood loss using PFNA versus IT. The dual-screw design of IT, which requires placement of an additional screw, may explain the greater fluoroscopy and operating times. Increased blood loss could be directly linked to longer operating times with IT or result from additional drilling for the second screw. However, Seyhan *et al.* indicate the most time-consuming part of the IT operation to be determining a correct entry point [15]. Furthermore, Wu *et al.* report that the IT device is more challenging to insert into smaller bone cavities because of its trapezoidal proximal nail end; the increased manipulation during this process could explain the increased intraoperative and fluoroscopy time and blood loss [16]. Despite the favorable intraoperative parameters of PFNA and Gamma3, which may influence the surgeon's decision on fixation device, it is important to note that intraoperative outcomes should be viewed within a wider context, alongside longer-term parameters. The absolute values for intraoperative parameters (Table 2) remain within reasonable clinical limits for the IT group and may be acceptable in light of better long-term fixation outcomes.

The findings from this review suggest that IT has several superior postoperative fixation outcomes compared with single-screw devices. Wu *et al.* and Zhang *et al.* reported significantly higher rates of femoral shaft fractures with PFNA and Gamma3 nails [16,19,21]. Femoral fractures occur at the tip of the nail insertion into the femoral shaft [23] and as a result of poor contact with the intramedullary wall [13]. IT has a tapered design that is hypothesized to reduce the concentration of stress around the nail tip, thereby reducing the risk of femoral fracture [19]. Additionally, Zhang *et al.* found on revision surgery that looseness of distal locking screws can contribute to femoral shaft fractures [19]. The results with regard to lateral cortex fractures were inconclusive, with Zhang *et al.* reporting higher rates of lateral cortex fractures in the PFNA group (p = 0.044) [19] and Yu *et al.* finding higher rates in the IT group (p = 0.045) [13].

Furthermore, screw migration was noted in some studies to be lower in IT patients compared with PFNA patients [12,23]. Several features of the IT design may provide resistance to screw migration, including superior fracture compression and a larger screw diameter compared with PFNA; Seyhan *et al.* also suggested that superior IT compression reduces screw migration [15]. Notably, there are a limited number of studies in the literature comparing screw migration rates between IT and Gamma3. The incidence of cut-out is also lower with IT compared with PFNA and Gamma3. Takigami* et al.* established several factors that influence the risk of cut-out: implant position, tip-to-apex distance (TAD), fracture reduction and time to weight-bearing [26]. The dual-screw design of IT is thought to provide better rotational stability and resist excess load upon weight-bearing compared with single-screw devices, thus reducing the risk of cut-out [12,13]. There are, however, fracture-related factors that can also determine cut-out rates; Gavaskar *et al.* emphasized the importance of categorizing fractures based on AO classification, as complication rates are higher in complicated fractures [27]. Seyhan *et al.* also noted that the skill of the surgeon impacted cut-out, with higher rates in inexperienced surgeons [15]. Cut-out rates have been shown to decrease as a result of learning curve in other long-term studies [24]. The variability in surgical experience across the studies was not documented and is therefore a limitation of this study.

Although IT appears to demonstrate favorable implant failure outcomes compared with PFNA and Gamma3, it is difficult to draw valid conclusions from the authors' data, and there is a need for longer-term follow-up in future studies. Although Serrano *et al.* demonstrated significantly higher rates of implant failure with Gamma3 (p = 0.007) [25], the difference in varus collapse was nonsignificant in the longer-term study by Hopp *et al.* [17]. Superior compression with IT may prevent uncontrolled shortening and subsequent varus collapse. In a review by Nherera *et al.*, the authors suggest that intramedullary nail length could influence rates of implant failure [9], although Serrano *et al.* argue that shortening and varus collapse are due to proximal fixation and not nail length [25]. The researchers found that proximal femoral shortening over 5 mm led to worse functional outcomes in patients, although no acceptable cut-off has been determined in the literature. Zhang *et al.* report that factors associated with implant failure and subsequent surgical revision are early weight-bearing, inadequate intraoperative positioning and poor fracture reduction [19]. Surgical technique and postoperative protocol are important and perhaps overlooked factors that can greatly influence the effectiveness of intramedullary nails.

Additionally, IT patients show lower rates of postoperative pain compared with single-screw device patients. Zhang *et al.* attributed this to the distal bifurcation of the IT nail causing fewer perioperative fractures and, subsequently, reduced pain [19]. This may also contribute to lower rates of cut-out within the IT group [9]. Although Su *et al.* and Berger-Groch *et al.* found increased short-term pain in Gamma3-treated patients, 5-year follow-up revealed no significant differences in pain [18,24]. Notably, there was a high rate of loss to follow-up, which may affect interpretation of the results. Postoperative pain is a difficult outcome to assess and compare across studies because of the subjective nature of pain, variation in prescribed analgesia and different assessment tools used in studies. This leads to difficulty in drawing reliable conclusions on postoperative pain benefits with IT. Although postoperative outcomes in IT-treated patients were largely favorable, studies evaluating time to union and cases of malunion showed minimal differences between IT and PFNA and Gamma3 nails. Notably, the incidence of malunion and nonunion was low across studies and therefore may not have been sensitive in detecting significant differences.

Some studies in this review discussed the utility of TAD in determining postoperative complications in intertrochanteric fractures. Zhang *et al.* found no difference in TAD between failed and successful implant surgery [21]. The researchers found that only 87% of patients with radiological reduction had a TAD <25 mm, and patients with implant failure had a TAD <19 mm [21]. TAD alone may be insufficient in predicting favorable outcomes, and the researchers suggest that bone quality may also impact the use of TAD [21]. A study by Nikoloski *et al.* argued that TAD <25 mm was too generous, and that this should be revised to <20 mm with PFNA to prevent axial migration [28]. A lower TAD limit is equally debated within the literature. A biomechanical study from Lenz *et al.* demonstrated no significant differences in number of cycles to failure between cadavers with normal (20 mm) and reduced (6 mm) TAD; however, the clinical relevance is yet to be determined [29]. Each intramedullary nailing system may have unique TAD requirements, as opposed to the one-size-fits-all approach used today. Although TAD is a useful parameter for predicting outcomes, further research is required to look at device-specific TAD and to identify if modified TAD criteria would be of benefit in select patient groups.

Choice of implant in patients with intertrochanteric fractures may also require consideration of bone quality. Many hip fractures are often associated with underlying osteoporosis, which can complicate surgery because of poor bone quality [19]. Zhang *et al.* hypothesized that there is a greater responsibility of the nail to support weight-bearing in osteoporotic patients, as poorer quality bone is unable to effectively distribute load [19]. Osteoporosis can cause greater postoperative complications, such as cut-out and implant failure, because of microfractures introduced during surgery [19]. PFNA has been stipulated to be more effective in frail patients with multiple comorbidities [12] because of its favorable intraoperative parameters and ability to provide better compaction compared with IT [13]. Further studies are required to stratify osteoporotic patients by disease severity to determine optimum treatment in varying patient groups.

This study supports the work of Nherera *et al.* and Ma *et al.* evaluating the effectiveness of IT versus PFNA and Gamma3 nails [9,30]. However, the authors of this study recognize several limitations. The review included a relatively small sample size of just 14 studies, of which only five compared IT and Gamma3. There was study heterogeneity in patient demographics, anesthesia use, postoperative physiotherapy and study design. The majority of included studies were retrospective or prospective cohort studies, and there is a need for further high-level evidence in the literature. However, included papers were thoroughly screened and were deemed to be of high quality, therefore minimizing the impact of these limitations. Studies also showed variability in follow-up times, ranging from 6 months to 5 years, and greater follow-up is required to assess the long-term viability of internal fixation devices and their impact on quality of life. Furthermore, some studies did not stratify between stable and unstable intertrochanteric fractures. Although outside the remit of this review, another important factor to consider is the cost–effectiveness of IT compared with PFNA and Gamma3 nails.

## Conclusion

The current literature suggests that there is no clear advantage of IT over PFNA and the Gamma3 nailing system. Although the use of IT demonstrated favorable outcomes with regard to postoperative pain, cut-out and screw migration, no differences were found in HHS and bone union. Intraoperative parameters of fluoroscopy time and operating time favored the single-screw devices over IT, with blood loss and length of hospital stay significantly lower in the PNFA group compared with the IT group. These findings can be used alongside clinical judgment to determine optimum treatment for patients. Further long-term randomized controlled trials are required to compare the benefits and drawbacks of IT versus other cephalomedullary nailing devices.

## Future perspective

With life expectancy predicted to increase, hip fractures will remain a significant cause of morbidity and mortality in the aging population, and research must be conducted to optimize treatment strategies for this cohort. We propose a shift toward a ‘tailor-made’ strategy for separate patient groups, taking parameters such as bone quality and fracture pattern into account. Future studies may also wish to evaluate the utility of bone quality and TAD as predictive markers for specific postoperative complications. Research is also required to determine acceptable TAD ranges for individual nailing devices, as TAD is unlikely to be a one-size-fits-all solution. Finally, studies are required to evaluate the cost–effectiveness of different cephalomedullary devices.

**Executive summary**

- Hip fractures remain a significant cause of morbidity and mortality within healthcare systems, with an estimated global incidence of 1.6 million fractures annually.
- Certain subtypes of unstable intertrochanteric fractures are surgically treated with the use of intramedullary nailing devices.
- Assessment of clinical and radiological outcomes reveals no clear advantage of TRIGEN™ InterTAN™ (IT) over the single-screw proximal femoral nail antirotation (PFNA) and Gamma3 nailing system.
- The results of the study should be used in conjunction with clinical judgment to determine optimum treatment for patients requiring surgical treatment of hip fractures.
